# The Risk of Cross Infection in the Emergency Department: A Simulation Study

**DOI:** 10.1017/ice.2018.61

**Published:** 2018-04-16

**Authors:** Vicki Stover Hertzberg, Yuke A. Wang, Lisa K. Elon, Douglas W. Lowery-North

**Affiliations:** 1 Nell Hodgson Woodruff School of Nursing, Emory University, Atlanta, Georgia; 2 Department of Biostatistics and Bioinformatics, Emory University, Atlanta, Georgia; 3 Department of Emergency Medicine, Emory University, Atlanta, Georgia

## Abstract

**OBJECTIVES:**

The risk of cross infection in a busy emergency department (ED) is a serious public health concern, especially in times of pandemic threats. We simulated cross infections due to respiratory diseases spread by large droplets using empirical data on contacts (ie, close-proximity interactions of ≤1m) in an ED to quantify risks due to contact and to examine factors with differential risks associated with them.

**DESIGN:**

Prospective study.

**PARTICIPANTS:**

Health workers (HCWs) and patients.

**SETTING:**

A busy ED.

**METHODS:**

Data on contacts between participants were collected over 6 months by observing two 12-hour shifts per week using a radiofrequency identification proximity detection system. We simulated cross infection due to a novel agent across these contacts to determine risks associated with HCW role, chief complaint category, arrival mode, and ED disposition status.

**RESULTS:**

Cross-infection risk between HCWs was substantially greater than between patients or between patients and HCWs. Providers had the least risk, followed by nurses, and nonpatient care staff had the most risk. There were no differences by patient chief complaint category. We detected differential risk patterns by arrival mode and by HCW role. Although no differential risk was associated with ED disposition status, 0.1 infections were expected per shift among patients admitted to hospital.

**CONCLUSION:**

These simulations demonstrate that, on average, 11 patients who were infected in the ED will be admitted to the hospital over the course of an 8-week local influenza outbreak. These patients are a source of further cross-infection risk once in the hospital.

*Infect Control Hosp Epidemiol* 2018;39:688–693

The risk of cross infection in a busy emergency department (ED) is a serious public health concern, especially during a pandemic threat. The importance of this risk was demonstrated dramatically during the 2003 severe acute respiratory syndrome (SARS) epidemic, in which 128 cases of SARS could be directly or indirectly linked to exposure to a SARS patient who sat for hours in the busy ED of a community hospital awaiting assignment to a hospital bed.^1^ More recently, the presentation of a patient infectious with Ebola virus disease to an ED in Dallas, Texas, resulted in a need to monitor more than 180 individuals, many of them hospital personnel, who were in close contact with this patient or with 2 nurses who became infected after exposure to this patient.^2^

Understanding the dynamics of cross infection in the ED will facilitate the development of improved mitigation efforts. To fully assess the risk of cross infection, one must not only understand the disease itself but also the complex spatial and social environment of the ED in which cross infection occurs. In the past, many simulation models have used random mixing patterns of all people present to quantify infections.^3^ ^–^ ^9^ However, for many situations, random mixing is not a good model, and for this reason, real-time location systems using technology such as radiofrequency identification (RFID), ultrasound, or infrared tagging are being increasingly used to empirically determine such mixing patterns.^10^ ^–^ ^12^

To this end, we undertook a study of close-proximity interactions (contacts) among patients and healthcare workers (HCWs) in a busy ED.^13^ We used these data to construct the social-contact networks we used to simulate the transmission of infection from 1 infectious individual present in the ED during a 12-hour shift. Our objectives were to quantify infectious disease risks due to contacts and to determine whether individual characteristics are associated with differential patterns in the risk of infection.

## METHODS

### Study Design

Data on contacts that occurred ≤1 m distance between patients and staff in the busy ED of a large urban hospital were collected using an RFID system described elsewhere.^13^ This ED has 25,000 square feet and 31 beds, with annual census exceeding 57,000, of whom more than 14,000 patients are admitted. The ED has a modern design with centralized workspaces for staff and walled patient treatment rooms. The Emory University Institutional Review Board reviewed and approved this study. The data we collected about the contacts were the study IDs of the 2 individuals involved and length of contact. From the study IDs, we were able to link to other individual characteristics.

Briefly, we had planned to observe contacts among patients and staff during two 12-hour shifts per week for 1 year. As implemented, we observed 293,181 contacts of 4,732 patient and 85 staff participants during 81 shifts during the study year (July 1, 2009, to June 30, 2010). In this study, we restricted our analysis to data from the first 6 months of the study (35 shifts). We restricted analysis to this subset of shifts because examination of participation by patients and staff across the year showed a significant decline. We attributed staff participation decline to a system failure that did not alert us to battery depletion in permanent tags worn by staff. No similar physical reason for the decline in patient participation was observed; thus, we attributed it to waning abilities of the research team to keep up with a task that was too large for them. Biases in estimates of measures of interest may have resulted from missing individuals and their concomitant contacts. We restricted analyses to shifts in the first 6 months of our observation period because the decline in staff participation started at the beginning of the second half of the year. The observations included here should not be biased by the presence of missing data.

### Simulation Modeling

For each pair of individuals involved in a contact, we calculated the total duration for all of their contacts during a shift. We then used this duration as input for the simulation model. Specifically, we modeled the probability of infection for a contact with an exponential distribution function on the duration of the interaction. We assumed a common risk for all individuals present, as would be the case for a novel infectious agent for which no vaccine exists. Specifically, we were interested in staff role, patient chief complaint, and patient arrival mode. We also assumed that all ED occupants other than the infectious source were disease free at the start of the shift. In addition, because the ED is an important source of hospital admissions (ie, with ~40% of admissions nationally stemming from an ED visit),^14^ we were interested in patient ED disposition status, that is, whether they were admitted to the hospital or not.

For purposes of this study, we assumed a susceptible–infected (SI) model with the probability of infection following an exponential distribution such that the probability of an infection in 1 minute was 0.007. We did not consider a full susceptible–infected–recovered (SIR) model because our limited period of observation did not allow us to determine whether an exposed participant became infectious after an appropriate incubation period. This parameterization was drawn from the observed attack rate of influenza over 3 hours in a commercial airliner.^15^ We considered each participant in all shifts as a possible single source of infection, and we simulated infection transmission 10,000 times for these individuals.

### Data Analysis

We computed the number of expected infections per shift (EIs) by averaging how many times a given participant in a given shift was “infected.” We then averaged the 10,000 simulations over all participants in all shifts. Patients were classified according to arrival mode (ie, by emergency medical services [EMS] or not), chief complaint as categorized by the ESSENCE criteria,^16^ and discharge disposition status (ie, admitted to the hospital or not). The HCWs were classified according to their role (ie, provider, nurse, or staff). From the simulation results, we were able to determine the percent infected for each participant; we then cross classified him or her according to the factors of interest and calculated summary statistics for the categories of participants.

## RESULTS

In the 6-month observation period, we made 1,263 observations of 85 distinct HCWs, and 2,374 patient encounters were observed. Overall, 45,877 contacts were observed. Table 1 provides descriptive statistics regarding the length of these contacts. Because there is considerable variability in contact length, the statistics are also provided for each type of contact. Notably, HCW–HCW contacts tended to be much longer than either HCW–patient or patient–patient contacts.

**TABLE 1:** Descriptive Statistics Regarding Length of Contacts by Type

Contact Type	Median Contact Length, min	Interquartile Range, min	No. of Observations
Overall	5.7	(0.8–31.5)	45,877
Patient–patient	3.1	(0.5–13)	16,453
HCW–patient	2.9	(0.4–11.1)	14,117
HCW–HCW	38.6	(4.4–188.2)	15,307

NOTE. HCW, healthcare worker.

**FIGURE 1:**
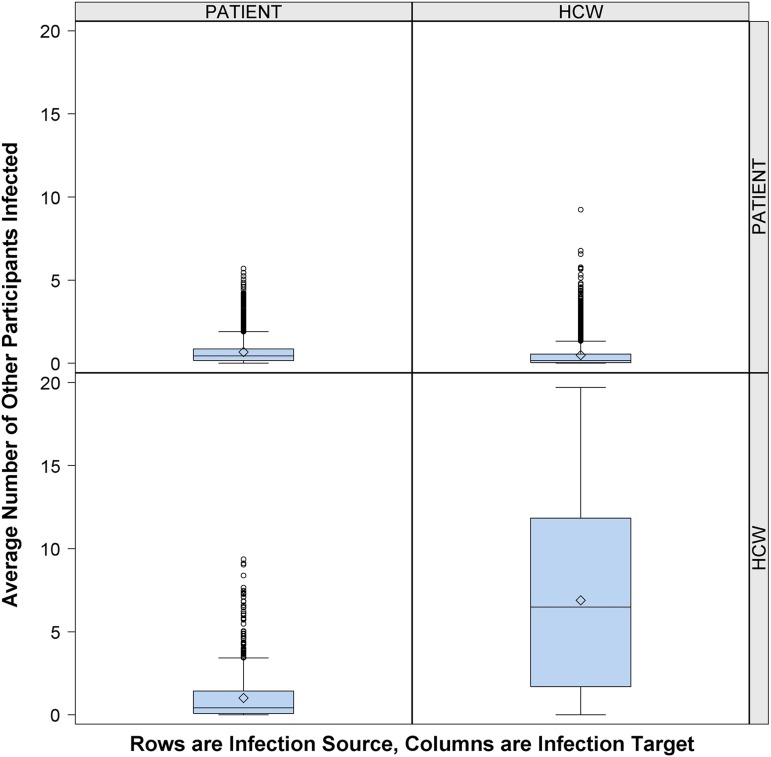
Risk of cross infection from infectious patient or infectious healthcare worker (HCW). Number of other participants infected (column) by 1 infectious person present in the emergency department (ED) (row), according to participant role (patient or HCW), over 10,000 simulations. Middle line indicates median; open diamond symbol is mean; upper and lower edges are placed at the 75th and 25th percentiles, respectively. Whiskers are placed at 1.5 times interquartile range beyond the 75th and 25th percentiles. Open circles indicate observations beyond the whisker values.

Figure 1 shows that the risk of cross infection was greater for other HCWs than for patients. In this context, an average of 0.4 patients and 6.5 other HCWs were infected due to contacts with an infectious HCW per 12-hour shift. In comparison, 0.4 other patients and 0.2 HCWs were infected due to contacts with an infectious patient per 12-hour shift.

**FIGURE 2:**
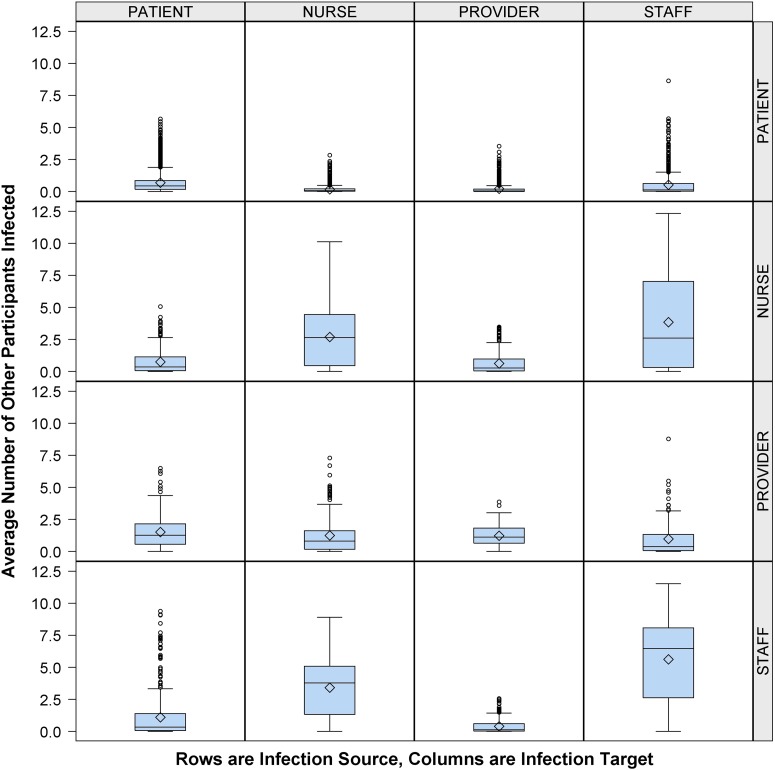
Average number of other participants infected by participant type, healthcare worker (HCW) role expanded. Number of other participants (column) infected by infectious person present in the emergency department (ED) (row), according to participant role (patient or HCW) with HCW role classified as provider, nurse, or staff. Middle line indicates median; open diamond symbol is mean; upper and lower edges are placed at the 75th and 25th percentiles, respectively. Whiskers are placed at 1.5 times the interquartile range beyond the 75th and 25th percentiles. Open circles indicate observations beyond the whisker values.

Having found that HCWs played a large role in cross transmission, we next evaluated the influence of staff role. Figure 2 shows that an infectious provider was more likely to infect a patient (1.3 EIs) or another provider (1.1 EIs) compared to nurses (0.8 EIs) or other staff (0.4 EIs). In comparison, infectious nurses and staff were more likely to infect their cohorts (2.6 nurse-to-nurse EIs vs 6.5 staff-to-staff EIs) or each other (2.6 nurse-to-staff EIs vs 3.8 staff-to-nurse EIs) than providers (0.8 provider-to-nurse EIs vs 0.4 provider-to-staff EIs).

Expected infections varied little across patient chief complaint categories, with the median EIs being <0.2 for all cross classifications. Most had median EIs<0.1 (Supplemental Figure 1). The same was true for a patient exposed to an infectious HCW and for an HCW exposed to an infectious patient.

We also categorized patients according to their arrival mode (ie, by EMS or not) (Supplemental Figure 2). Susceptible patients who arrived by EMS had modestly but consistently lower median EIs values than those who arrived by other means, regardless of the source of infection: EMS patients (0.12 vs 0.23), non-EMS patients (0.09 vs 0.30), caregiver (0.14 vs 0.28), provider (0.57 vs 0.87), or staff (0.05 vs 0.28).

Finally, we classified patients according to their ED disposition status (ie, admitted to hospital or not) (Supplemental Figure 3). On average, we detected 0.1 EIs among susceptible patients who were subsequently admitted to the hospital, regardless of infection source.

## DISCUSSION

This study is the first direct observation of contacts among patients and staff in the hospital ED environment and the first application of network science to such interactions over an extended period. It is also the first to examine differences in EIs by participant characteristics. As such, we have 2 major findings. First, we have shown that HCWs who report to work sick are the primary vectors for cross infection in the ED for respiratory diseases spread by large droplets. Furthermore, HCWs in the ED tend to view patients rather than their colleagues as the primary vectors for cross infection risk. The magnitude of the difference in EIs between infectious HCWs and infectious patients is surprising. Moreover, the differential among HCW roles was unanticipated; staff and nurses are more likely than providers or patients to infect others based only on the number and duration of their proximal interactions. Second, EMS arrivals are at lower risk of becoming infected than patients arriving by other modes. This observation is somewhat surprising; there is often a swarm of activity surrounding an EMS patient when he or she enters the ED, and this swarm might be expected to lead to more EIs.

Our study has several strengths. Many other infectious disease simulations^3^ ^–^ ^9^ assume random mixing patterns, while our study is based on empirical data with which the (nonrandom) mixing pattern is measured. In addition, we measured the duration of all contacts between individuals throughout a shift, allowing us to give a time-based rather than contact-based risk of infection. In contrast, many other studies of contacts using technology (eg, RFID, infrared motes) to determine mixing patterns have done so over comparatively short periods of time (1 day to 1 week).^10^ ^–^ ^12^ ^,^ ^17^ ^–^ ^19^ In comparison, we have measured shifts over many months. Finally, we are the first to apply these sophisticated modeling techniques to determine infection risk in the ED setting.

In this simulation, we incorporated only 1 infection rate based on a case study of influenza transmission.^15^ Our model assumed that all exposed individuals were equally susceptible and did not allow for differential probability of infection. Thus, our model does not allow for a vaccine-preventable infection. Also, some patients may be more likely to become infected because they are immunocompromised. In addition, HCWs may use personal protective equipment (PPE) or rigorous hand hygiene (HH) measures, decreasing the probability of cross infection. However, HCW adherence rates to HH protocols are generally low, varying from 40% to 60%.^20^ Moreover, HCWs are likely not using PPE and HH measures when interacting with one another. Our model also approximates the scenario in which infection is spread only via large droplets, not allowing for cross infection via aerosolized virus.^21^ Our model assumes a given infection rate (ie, probability of infection per unit time) based on influenza, but this rate can be varied up or down for infectious agents that are more or less likely to cause infection in a given period of time, for example, measles or the coronavirus associated with Middle Eastern respiratory syndrome. As these rates vary up or down, the numbers of infections resulting will likewise vary up or down in a multiplicative fashion.

Notably, the providers have different risk patterns than other HCW groups. We attribute these differential risks to the workflow in this ED. At the time of the study, the nurses and staff worked as teams, each with an assigned group of beds, whereas the providers worked throughout the ED.

Interestingly, we found no differential risk of cross infection between patients according to chief complaint categories. Possibly, the ESSENCE criteria do not differentiate on features that are related to contact times. We also found no differential risk associated with ED disposition status. Nonetheless, although 0.1 EIs for patients admitted to the hospital seems small on a per-shift basis, it would result in ~11 patients admitted over the course of an 8-week local outbreak of seasonal influenza, providing further opportunities for cross infection in other hospital areas.

The results of this simulation study reinforce our findings of differences in network connectivity measures between patients arriving via EMS versus patients arriving via other means.^22^ Here, there is a differential risk via patient transport category and HCW role. Risk of cross infection is greatest between non-EMS patients and staff, while risk of cross infection between EMS patients and staff is much lower than between EMS patients and either providers or nurses, and these are much less than risks between non-EMS patients and either providers or nurses. Intuitively, one would presume that patients arriving by EMS are typically of higher acuity than patients arriving by other modes; thus, they would need more HCW contact time to sort out their complex issues, contrary to what we observed. There are several possible reasons for these counterintuitive findings: (1) Patients arriving by EMS have a different geographical pathway of care compared to non-EMS patients. EMS patients are taken from the ambulance bay directly to a patient room, and most services (e.g., triage, phlebotomy, etc) come to them. Non-EMS patients typically stay in the waiting room between these same services until a provider is ready for them, at which point they go to a patient room. (2) Healthcare workers deliver some services in the ED to non-EMS patients that EMS personnel deliver to EMS patients *en route* (eg, obtaining medical history, starting intravenous drips, starting oxygen delivery). Thus, the time that HCWs initially need with an EMS patient at the beginning of the admission is curtailed. (3) Because EMS patients are more acutely ill, they are more likely to be admitted to the hospital than non-EMS patients. The time spent at the end of the visit is much less because there is no need for HCW involvement in discharge activities such as getting the patient dressed, getting the patient into a wheelchair, or giving discharge instructions.

In addition, patients arriving with respiratory infection do not appear to differ substantially in rates of infection of other ED occupants. This may be due to observation of infection control measures oriented to patients. These measures, oriented toward patients, center around signage encouraging cough etiquette, whereas measures oriented toward HCWs include mandatory vaccination policies and use of PPE and HH during contacts with patients. Our results reinforce the importance of mandatory vaccination policies for HCWs and policies prioritizing HCWs for receiving vaccines as developed in response to novel infections. We speculate that there is considerable social pressure for HCWs to work, especially in epidemic periods. This behavior must change. In addition, HCWs should use PPE and HH during periods with potential for contacts among themselves to reduce cross-infection risk. As new EDs are built and existing EDs renovated, architectural and engineering solutions must be found to artificially create social distance in areas with high HCW–HCW contact potential. Our findings present opportunities to reimagine infection control in this environment.

Although we have identified differential risks for HCWs depending on role and patient arrival mode, we have not defined the geographical areas of the ED in which the HCW–HCW contacts have occurred nor the geographical pathways of care for patients and resultant contacts. Thus, future work should characterize the geographical distribution of these contacts and of risk of cross infection. Such understanding will pave the way for structural and functional countermeasures for cross infection.

## Supplementary material

For supplementary material accompanying this paper visit http://dx.doi.org/10.1017/ice.2018.61. click here to view supplementary material
