# Supplementary material for: The Risk of Cross Infection in the Emergency Department: A Simulation Study
Source: Infect Control Hosp Epidemiol. 2018 Apr 16;39(6):688–93. doi: 10.1017/ice.2018.61 (PMC6088782; doi:10.1017/ice.2018.61)

**Supplementary Materials**

**for**

**“The Risk of Cross Infection in the Emergency Department: A Simulation Study” by Hertzberg, Wang, Elon, and Lowery-North**

**Methods**

Data on contacts ≤ 1 m among patients and staff in the busy ED of a large urban hospital were collected using an RFID system as described elsewhere.[^3^](#_ENREF_3) The protocol was reviewed and approved by the Emory University Institutional Review Board. Briefly, we had planned to observe contacts among patients and staff during two 12-hour shifts per week for one year. As implemented, we observed 293,181 contacts of 4,732 patient and 88 staff participants during 81 shifts during the study year (1 July 2009 – 30 June 2010). In this paper we restrict our analysis to data from the first 6 months of the study (35 shifts). Data limitations that led to restricted analysis on a subset of shifts are as follows: Examination of participation by patients and staff across the year showed a significant decline. We attribute staff participation decline to a system failure that did not alert us to battery depletion in permanent tags worn by staff. There is no similar physical reason for decline in patient participation, thus we attribute it to waning abilities of the research team to keep up with a task that was too large for them alone. Biases in estimates of measures of interest can result from missing individuals and their concomitant contacts. Thus we restricted analyses to shifts in the first 6 months of our observation period since the decline in staff participation starts at the beginning of the second half of the year and thus these observations should not be biased by the presence of missing data.

For each pair of individuals involved in a contact, we calculated the total duration for all of their contacts during a shift. We then used this duration as input for the simulation model. In particular we model the probability of infection for a contact with an exponential distribution function on the duration of the interaction. We assumed a common risk for all individuals present such as could be the case for a novel infectious agent for which no vaccine exists. Specifically we were interested in staff role, patient chief complaint, and patient arrival mode. In addition, since the ED is an important source of hospital admissions, with approximately 40% of admissions nationally stemming from an ED visit,[^22^](#_ENREF_22) we were interested in patient ED disposition status, i.e., whether they were admitted to the hospital or not. For purposes of this study we assumed a susceptible – infected (SI) model with the probability of infection followed an exponential distribution such that the probability of an infection in one minute equals 0.007. We did not consider a full SIR model since our limited period of observation does not allow us to determine if an exposed participant becomes infectious after an appropriate incubation period. This parameterization was drawn from the observed attack rate of influenza in a commercial airliner, in which 38 of 53 passengers and crew were infected with influenza over the course of 3 hours of exposure to an infectious passenger.[^19^](#_ENREF_19) We considered each participant in all shifts as a possible single source of infection, and simulated infection transmission 10,000 times for these individuals. From these data we were able to determine the percent infected for each participant, then cross-classify her/him according to the factors of interest, presenting summary statistics for the categories of participants.

Supplemental Figure 1. Average Number of Other Participants Infected, Patient Syndrome Expanded. Number of other participants (column) infected by infectious person present in the ED (row), according to participant role (patient or HCW) with patients classified by chief complaint as categorized by CDC/ESSENCE criteria. Middle line indicates median, open diamond symbol is mean, upper and lower edges are placed at 75^th^ and 25^th^ percentiles, respectively, whiskers are placed at 1.5 times interquartile range beyond the 75^th^ and 25^th^ percentiles, and open circles indicate observations beyond the whisker values.


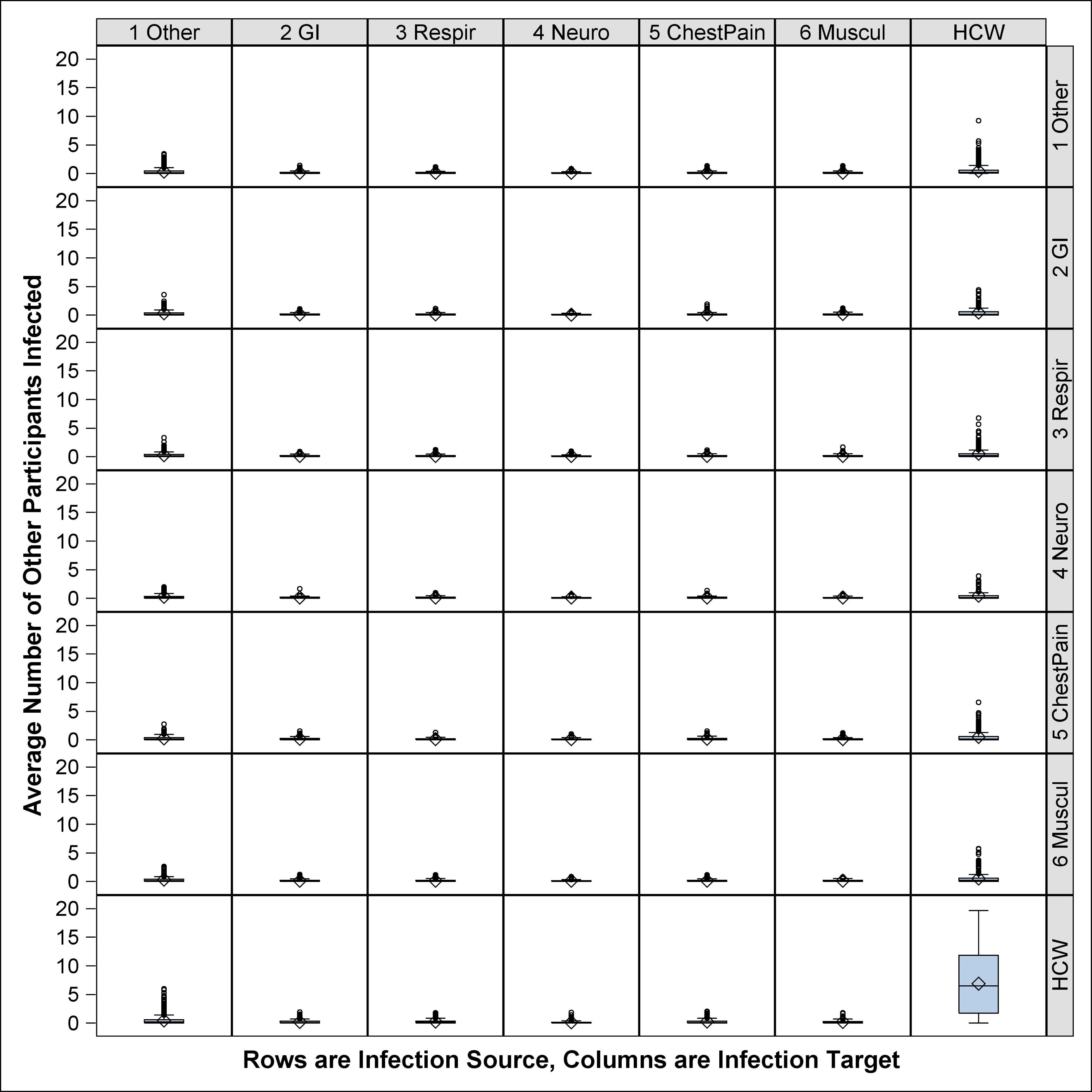


Supplemental Figure 2. Average Number of Other Participants Infected, Patient Arrival Mode and Staff Role Expanded. Number of other participants (column) infected by one infectious person (row) present in the ED, according to participant role (patient or HCW) with patients further categorized by arrival mode and HCWs further categorized by role. Middle line indicates median, open diamond symbol is mean, upper and lower edges are placed at 75^th^ and 25^th^ percentiles, respectively, whiskers are placed at 1.5 times interquartile range beyond the 75^th^ and 25^th^ percentiles, and open circles indicate observations beyond the whisker values.


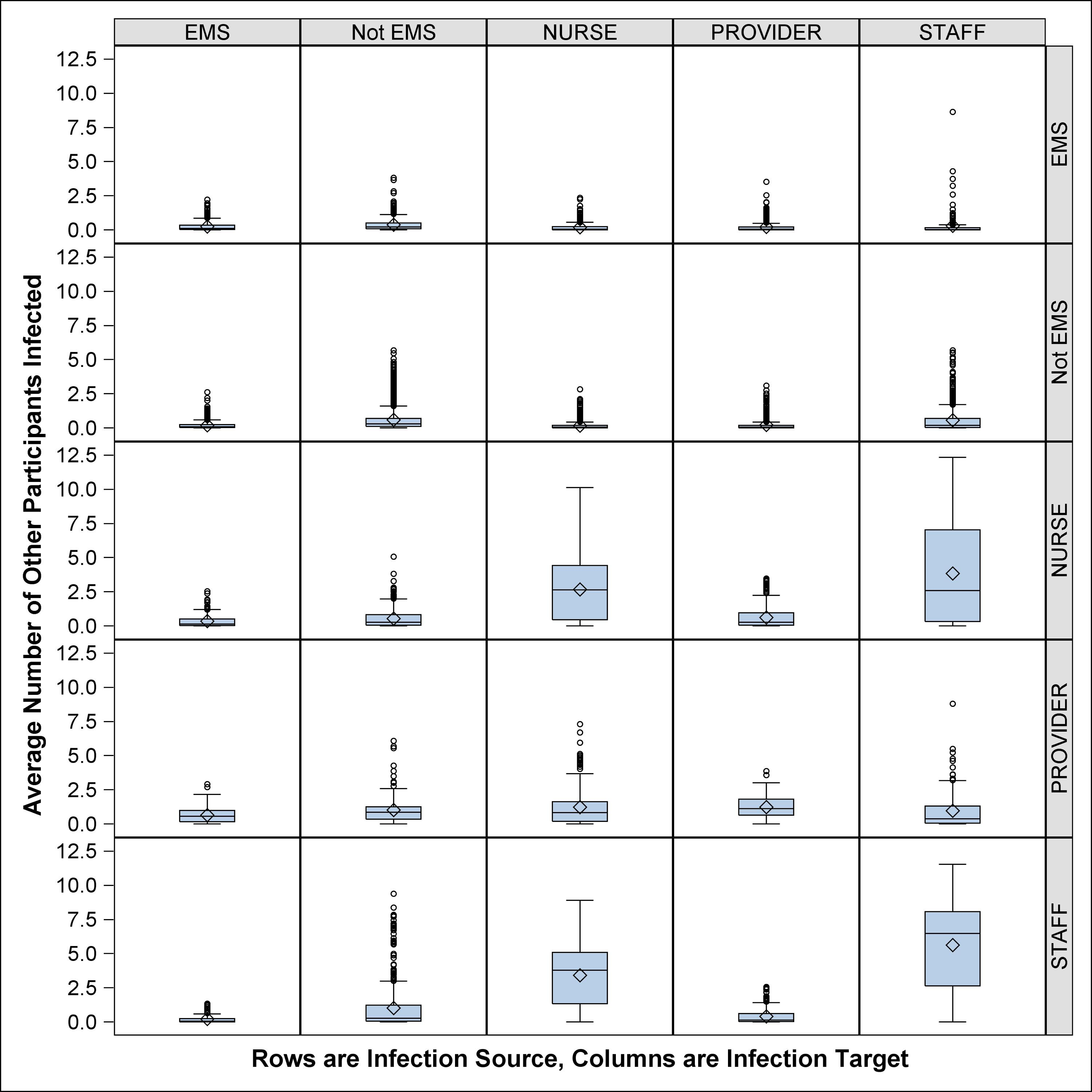


Supplemental Figure 3. Average Number of Other Participants Infected, Patient Discharge Status Expanded. Number of other participants (column) infected by one infectious person (row) present in the ED, according to participant role with patients further categorized by discharge status (admitted to hospital or not). Middle line indicates median, open diamond symbol is mean, upper and lower edges are placed at 75^th^ and 25^th^ percentiles, respectively, whiskers are placed at 1.5 times interquartile range beyond the 75^th^ and 25^th^ percentiles, and open circles indicate observations beyond the whisker values.


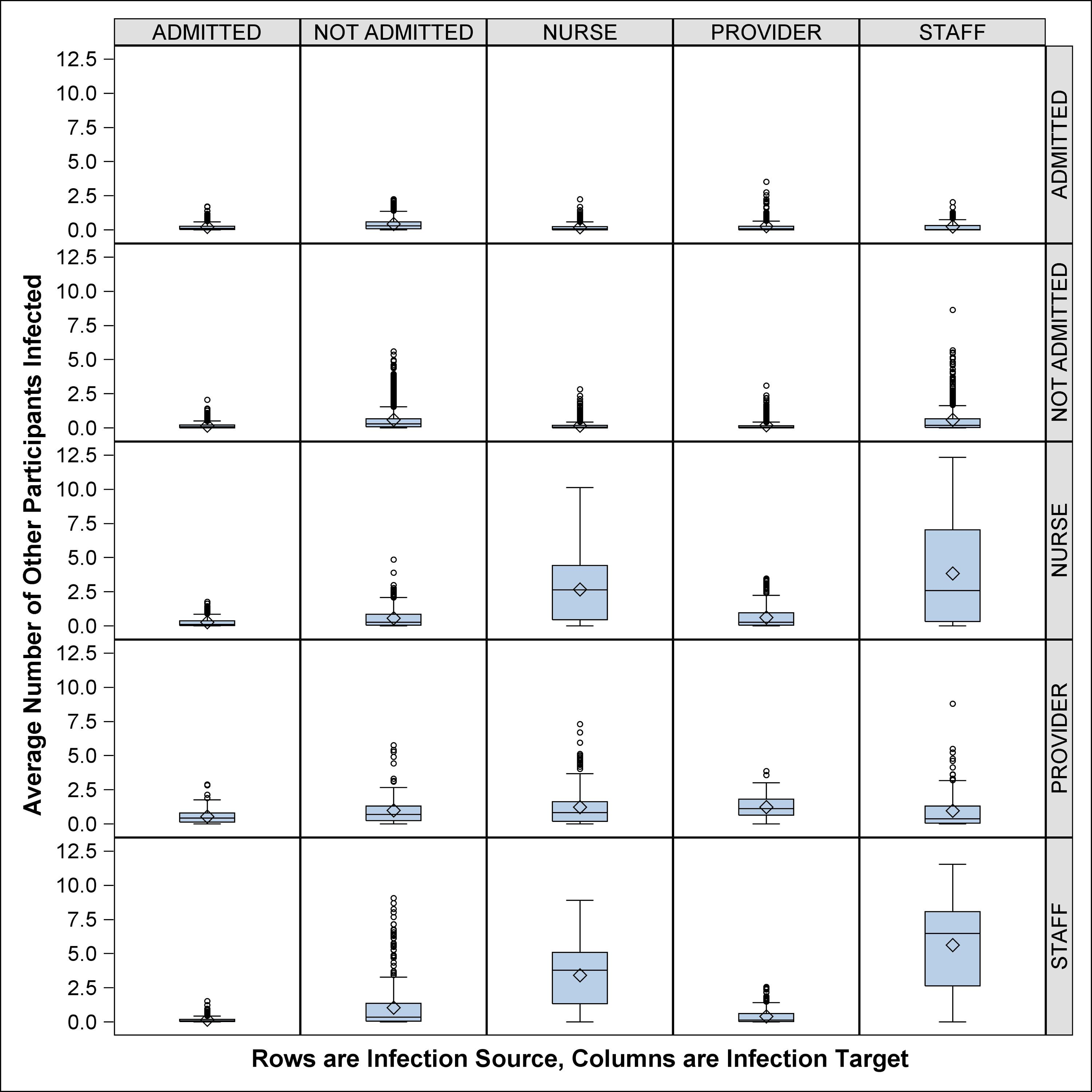

Supplement: Supplementary file 1 [file S0899823X18000612sup001.docx]
